# Heidelberg Neuro-Music Therapy Enhances Task-Negative Activity in Tinnitus Patients

**DOI:** 10.3389/fnins.2017.00384

**Published:** 2017-07-07

**Authors:** Christoph M. Krick, Heike Argstatter, Miriam Grapp, Peter K. Plinkert, Wolfgang Reith

**Affiliations:** ^1^Department for Neuroradiology, Saarland University HospitalHomburg, Germany; ^2^German Research Centre for Music Therapy ResearchHeidelberg, Germany; ^3^Department of Otorhinolaryngology, Head and Neck Surgery, University Hospital for Ear, Nose, and Throat, University of HeidelbergHeidelberg, Germany

**Keywords:** fMRI, tinnitus, Heidelberg Model of Music Therapy, RSN, neuroplasticity, recent-onset tinnitus, precuneus

## Abstract

**Background:** Suffering from tinnitus causes mental distress in most patients. Recent findings point toward a diminished activity of the brain's default-mode network (DMN) in subjects with mental disorders including depression or anxiety and also recently in subjects with tinnitus-related distress. We recently developed a therapeutic intervention, namely the Heidelberg Neuro-Music Therapy (HNMT), which shows an effective reduction of tinnitus-related distress following a 1-week short-term treatment. This approach offers the possibility to evaluate the neural changes associated with the improvements in tinnitus distress. We previously reported gray matter (GM) reorganization in DMN regions and in primary auditory areas following HNMT in cases of recent-onset tinnitus. Here we evaluate on the same patient group, using functional MRI (fMRI), the activity of the DMN following the improvements tinnitus-related distress related to the HNMT intervention.

**Methods:** The DMN activity was estimated by the task-negative activation (TNA) during long inter-trial intervals in a word recognition task. The level of TNA was evaluated twice, before and after the 1-week study period, in 18 treated tinnitus patients (“treatment group,” TG), 21 passive tinnitus controls (PTC), and 22 active healthy controls (AC). During the study, the participants in TG and AC groups were treated with HNMT, whereas PTC patients did not receive any tinnitus-specific treatment. Therapy-related effects on DMN activity were assessed by comparing the pairs of fMRI records from the TG and PTC groups.

**Results:** Treatment of the TG group with HNMT resulted in an augmented DMN activity in the PCC by 2.5% whereas no change was found in AC and PTC groups. This enhancement of PCC activity correlated with a reduction in tinnitus distress (Spearman Rho: −0.5; *p* < 0.005).

**Conclusion:** Our findings show that an increased DMN activity, especially in the PCC, underlies the improvements in tinnitus-related distress triggered by HNMT and identify the DMN as an important network involved in therapeutic improvements.

## Introduction

Suffering from tinnitus causes mental distress in most patients. Especially in situations with reduced external sensory input (e.g., at night, in silent rooms), the tinnitus percept comes to the fore and can cause emotional distress (Møller, 2016). Focused attention on the tinnitus can trigger sleeping disorders and impair concentration, regeneration as well as mood (Husain, 2016).

Over the past 15 years, great effort has been made to clarify the origin and tinnitus sensitivity of the so-called “task-negative activation” (TNA) during the brains “resting state” when less sensory input needs to be processed (Raichle et al., 2001; Buckner et al., 2008, 2013). Numerous experiments using functional Magnetic Resonance Imaging (fMRI) contrasted resting state brain activity during phases of less salient stimulation (TNA) vs. brain activity during active task performance (task-positive activation; Shulman et al., 1997; Buckner, 2013). Doing nothing also activates these regions of the brain's resting state (Fox et al., 2005). Whereas, the task-positive activity usually varies with study design, the task negative activity shows a very consistent task-independent pattern (Gusnard and Raichle, 2001). These TNAs in phases of no activity converge with the resting state network (RSN) in areas of the brain's “Default-mode Network” (DMN) comprising the medial prefrontal cortex (MPF), the posterior cingulate cortex (PCC) with an extension to the precuneus, and the lateral parietal cortex (LP; Raichle, 2015).

The functional meaning of the DMN is assigned to mental relaxation, introspective and autobiographical self-awareness, emotional regulation, and internal mental projections between past and future (Gusnard et al., 2001; Andrews-Hanna, 2012). The intrinsic DMN activation has been shown to be reduced in patients with psychiatric disorders (Broyd et al., 2009; Sripada et al., 2012) but also in patients experiencing tinnitus-related distress (De Ridder et al., 2014; Husain and Schmidt, 2014; Lanting et al., 2016; Leaver et al., 2016). However, opposite findings with respect to recent-onset tinnitus have been observed, though focused on the precuneus, too (Carpenter-Thompson et al., 2015). Thus, it seems consequential to further explore the relationship between precuneus and tinnitus distress.

Impaired resting state due to tinnitus perception influences the dorsal attention system which is related to top-down attention control during task execution (Husain, 2016). The impaired DMN function is also thought to contribute to the typical stress-related symptoms such as sleep disturbance, anxiety, depression, irritation, and concentration difficulties (Langguth, 2011; Schmidt et al., 2013; Vanneste and De Ridder, 2015; Møller, 2016).

Neural networks for tinnitus are present in auditory and non-auditory areas (De Ridder et al., 2014; Elgoyhen et al., 2015). Findings on TNAs and neural connectivity results from resting-state fMRI indicated that the PCC/precuneus region is also corrupted by tinnitus (Schmidt et al., 2013; Han et al., 2015; Vanneste and De Ridder, 2015; Lanting et al., 2016) due to its role in the brain's sleep control (Shannon et al., 2013) which in turn is impaired by tinnitus (Alster et al., 1993). However, the relationship between distress and precuneus involvement is still unclear. Following the observations of Lanting et al. (2016) and Schmidt et al. (2013), we argued that the state of mental rest may be corrupted by the tinnitus, since it induces a salient stimulus that in turn reduces DMN activation at rest. We measured task-positive and task-negative activity using fMRI in a visual word recognition task in tinnitus patients and healthy controls both treated with HNMT, as well as in untreated tinnitus patients. The levels of task-negative activity before and after the 1-week treatment period were contrasted to observe their specific alterations induced by HNMT. Since the HNMT has been shown to rapidly reduce the tinnitus distress, this design allowed us to observe the relationship between level of distress and TNAs. Our hypothesis argues that HNMT would allow tinnitus patients to reactivate their DMN after the treatment.

Recent findings from music therapy research revealed an inverse cortical reorganization in the DMN areas following a 1-week application of the Heidelberg Neuro-Music Therapy (HNMT): growth of gray matter (GM) density has been found in the PCC and the precuneus, suggesting a potential influence of HNMT onto DMN activity (Krick et al., 2015). Although, the neuroplastic changes can be deemed as an indirect indicator for a long-term alteration of the activity level, the best evidence for the activity behavior consists in examining the activity itself. This paper aims to evaluate changes in the DMN activity induced by HNMT.

## Materials and methods

### Participants

In this study, we included data from participants which were recorded in the context of a recent therapy control study where data from structural MRI measurements have already been reported (Krick et al., 2015). In this context, data for Blood Oxygen Level Dependency (BOLD) imaging were derived parallel to the structural measurements. However, whereas the previous study observed structural changes following HNMT longitudinally, the current study addressed changes in brain activity in the same participants. This convergence allowed complementary viewing both perspectives in the same brain regions.

Fifty patients with recent onset of tinnitus (between 6 and 12 weeks prior to the intervention) were invited to participate in the HNMT study after a treatment following the standard clinical protocol for acute tinnitus in the University Hospital for Ear, Nose, and Throat at the University of Heidelberg. Audiograms and psychological examination were conducted before the decision for study inclusion. All patients had age-appropriate hearing levels (audiograms are attached as Supplementary Material) and reported no otological or psychological co-morbidity. Severe hearing impairment was an exclusion criterion in this study. At the time of the pre-participation evaluation (T0), patients were randomly assigned to one of two groups: either a treatment group (TG) receiving a 5-day 1-week treatment with HNMT or a group of passive tinnitus controls (PTC) who did not receive any tinnitus specific treatment. The patient groups did not differ in biographical characteristics nor in tinnitus-related parameters (Table 1). For ethical reasons, PTC patients also received therapeutic intervention, but this was delivered subsequent to the study period. Participants in both groups were informed about MRI measurements and the noise level of the scanner. All participants were insured for any health impairment and accidents. They gave written informed consent in accordance with the Declaration of Helsinki. The study was approved by the ethical review board of Saarland (ID-number 111/11).

**Table 1:** Overview of participant groups and tinnitus parameters.

	**TG (*n* = 18)**	**PTC (*n* = 21)**	**AC (*n* = 22)**	**Statistics**
Tinnitus causation [acute hearing loss/noise trauma/distress/other] (*n*)	1/7/6/4	2/6/8/5		χ^2^_(*df* = 1)_ = 0.489 *p* = 0.484
Type of tinnitus [tonal/non-tonal] (n)	10/8	12/9		$\chi_{\left(df=1\right)}^{2}$ = 0.170 *p* = 0.680
Tinnitus frequency (Hz) [mean (*SD*)]	5,112 (2,382)	6,369 (3,193)		*t*_(**df** = 41)_ = −1.469 *p* = 0.154
Tinnitus localisation [right/left/bilateral/not determinable] (*n*)	5/7/4/2	4/8/5/4		χ^2^_(**df** = 1)_ = 0.146 *p* = 0.703
TQ score from initial anamnestic diagnostics [mean (*SD*)]	38.50 (15.4)	36.20 (16.82)		*t*_(**df** = 41)_ = 0.737 *p* = 0.465
Tinnitus duration up to initial anamnestic diagnostics (T_0_) (weeks) [mean (*SD*)]	5.10 (2.14)	4.63 (2.01)		*t*_(**df** = 41)_ = 0.567 *p* = 0.575
Tinnitus duration up to start of therapy (T_1_) (weeks) [mean (*SD*)]	8.14 (1.85)	8.10 (1.45)		*t*_(**df** = 41)_ = 0.082 *p* = 0.935
Patients' age (years) [mean (*SD*)]	43.9 (10.4)	42.6 (11.5)	38.9 (14.0)	Kruskal–Wallis test χ^2^_(**df** = 2)_ = 1.54 *p* = 0.464
Patients' sex [male/female] (n)	10/8	13/8	12/10	χ^2^_(**df** = 2)_ = 0.273 *p* = 0.873

Seven patients were excluded due to disappearance of tinnitus before the first MRI session. Two patients were excluded because of claustrophobia during the first MRI session. One patient was excluded due to image artifacts in the anatomical scan. Thus, the effective sample comprised 19 patients in the TG group and 22 patients in the PTC group. The mean delay between onset of tinnitus and first MRI session (T1) was 8.14 (*SD* 1.85) weeks in the TG group and 8.10 (*SD* 1.45) weeks in the PTC group [*t*_(*df* = 41)_ = 0.082; *p* = 0.935].

A group of 22 healthy participants were recruited to match the sex and age profile of the tinnitus patients. This group served as active controls (AC) as they also underwent HNMT as implemented in the TG group. Due to motion artifacts, two fMRI data sets had to be discarded from analysis (one from TG and one from PTC). Thus, the sample size comprised 61 participants in the present report in contrast to the original 63 reported in the preceding paper (Krick et al., 2015). The groups and the respective parameters are summarized in Table 1.

### Intervention (HNMT)

The study protocol consisted of nine consecutive sessions of individualized therapy, comprising acoustic training for frequency discrimination, auditory attention control tasks, and guided exercises for mindfulness and distress regulation. Therapy took place on 5 consecutive days (from Monday to Friday) with two therapy sessions per day (except Friday with one session). Music therapy can be divided into two main categories, receptive (music listening based) and active (music making). Each morning and each afternoon session lasted for 50 min, with 25 min of active music therapy and 25 min of receptive music therapy. Two trained music therapists carried out the therapy. One therapist performed the active modules, the other the receptive modules. The interventions were structured into treatment modules of directive counseling, habituation training, and stress management. A more detailed description can be found in Argstatter et al. (2014) and Grapp et al. (2013).

### Distress evaluation

Psychological complaints were assessed using the German version of the “Tinnitus Questionnaire” (TQ) as described by Goebel and Hiller (1998). This well-validated inventory comprises 52 items and records tinnitus related complaints. The items can be aggregated to variables representing the dimensions of mental distress: emotional and cognitive load, tinnitus duration, hearing impairments, sleep disturbance, and somatoform disorders. The global TQ-score ranges between the minimum score of 0 and the maximum score of 84, where high values indicate high tinnitus-related distress. Four levels of severity have been established for mild (0–30), middle (31–46), severe (47–59), and very severe (60–84) distress.

TQ scores were obtained at start of the study period (T1), and after the study week (T2). These two measurements were compared to quantify subjective alterations in the level of tinnitus-related distress over the study week.

The results of distress evaluation for the participants of the current study have been reported in the context of structural findings in Krick et al. (2015). In the TG and PTC groups, the initial TQ scores at T0 were similar at 38.50 ± 15.4 (*SD*) and 36.20 ± 16.82 (*SD*), respectively, [*t*_(*df* = 41)_ = 0.737; *p* = 0.465]. The HNMT application resulted in a significant difference in TQ scores (*T* = −5.7, *df* = 18, *p* < 0.0001) between the TG and PTC groups. In the TG group, the tinnitus distress score decreased by 17.7 TQ scale points (*SD* 13.6). In the PTC group, the TQ scores did not significantly change. The therapy effect on the subjective distress was further confirmed by a 2 × 2 repeated measures ANOVA (*df* = 1; *F* = 22.9; MSE = 1374; *p* < 0.00005).

### Imaging paradigm

DMN activity was assessed by means of event-related fMRI observing activation for rest conditions in contrast to a visual word recognition task. The task consisted in meaningful words (task condition) and non-sense strings (rest condition). The visual stimuli were presented by a projector behind the MR cabinet through a shielded window on a semitransparent screen. The screen was viewable over a mirror system placed on the top of the head coil. The rest condition consisted of non-sense letter strings for visual compensation, but no reaction was required from the participants. The length of the letter strings was matched for those of the meaningful words used.

Since in general the MRI technique is accompanied with a considerable noise emission, there was need for noise cancellation, especially for tinnitus sufferers. The visual presentation allowed for use of earplugs in addition to noise-cancelling headphones.

Each 24-min fMRI session comprised 960 stimuli, including 160 meaningful words and 800 non-sense strings. Stimuli were presented with a frequency of 40 stimuli per minute for continuous visual stimulation. Presentation time was each 1 s for words and non-sense strings. Participants were required to press a button when they recognized a meaningful word. Between two meaningful words, non-sense strings were presented. The participants were asked to monitor the non-sense strings without any action but to wait for the next meaningful word. For the purpose of analyzing the TNA, the brain response during the rather long inter-trial intervals (ITI) of 8.8 ± 7.65 s (*SD*) as task-negative periods was contrasted with the word recognition condition. Functional scan parameters of the BOLD imaging were set to time-to-repeat (TR) of 2.2 s, echo time (TE) to 30 ms, and a flip angle of 90°. Thirty slices of 3 mm thickness with a 0.75 mm gap to the next slice covered the whole brain. Each slice was scanned into a 94 × 94 matrix resulting in a voxel volume of 2 × 2 × 3 mm^3^. For each run, 655 scans were acquired including 4 preceding prescans for compensating the magnetization saturation effects. The prescans were later discarded from data analysis.

In addition, two high-resolution anatomical whole-brain scans were obtained at T1 and T2, using a Magnetization Prepared Rapid Acquisition of Gradient Echoes (MPRAGE; Mugler and Brookeman, 1990) sequence. These 3D brain images resulted in isometric voxel dimensions of 0.9 × 0.9 × 0.9 mm^3^.

Preprocessing and modeling of MRI measurements were executed according to standard procedures detailed in the software “Statistical Parametric Mapping” (SPM8, Wellcome Trust Centre for Neuroimaging, London). The preprocessing of the functional scans consisted of slice time correction, motion correction, segmentation of the anatomical scan and co-registration the resulting gray matter (GM) compartment to the mean image of corrected functional scans, co-registering the anatomical-image to the position of the functional mean image, determination of normalization parameters using the anatomical image, normalization of functional scans to the template of the Montreal Neurological Institute (MNI space), and final Gaussian smoothing using a 8 mm radius in each direction.

Both the functional and the anatomical MRI sequences were conducted twice, before (T1) and after (T2) the study period. During this time span, participants from the TG and AC groups were treated with HNMT. The first-level task-negative activity of both time points per participant was included in a second-level “flexible factorial model” (2 × 2 ANOVA) as implemented in SPM. This model used “subject,” “time,” and “group” as factors with an assumption of interaction between “time” and “group” to predict the influence of HNMT on the subjects' brain activity.

## Results

### DMN activity

**Figure 1:**
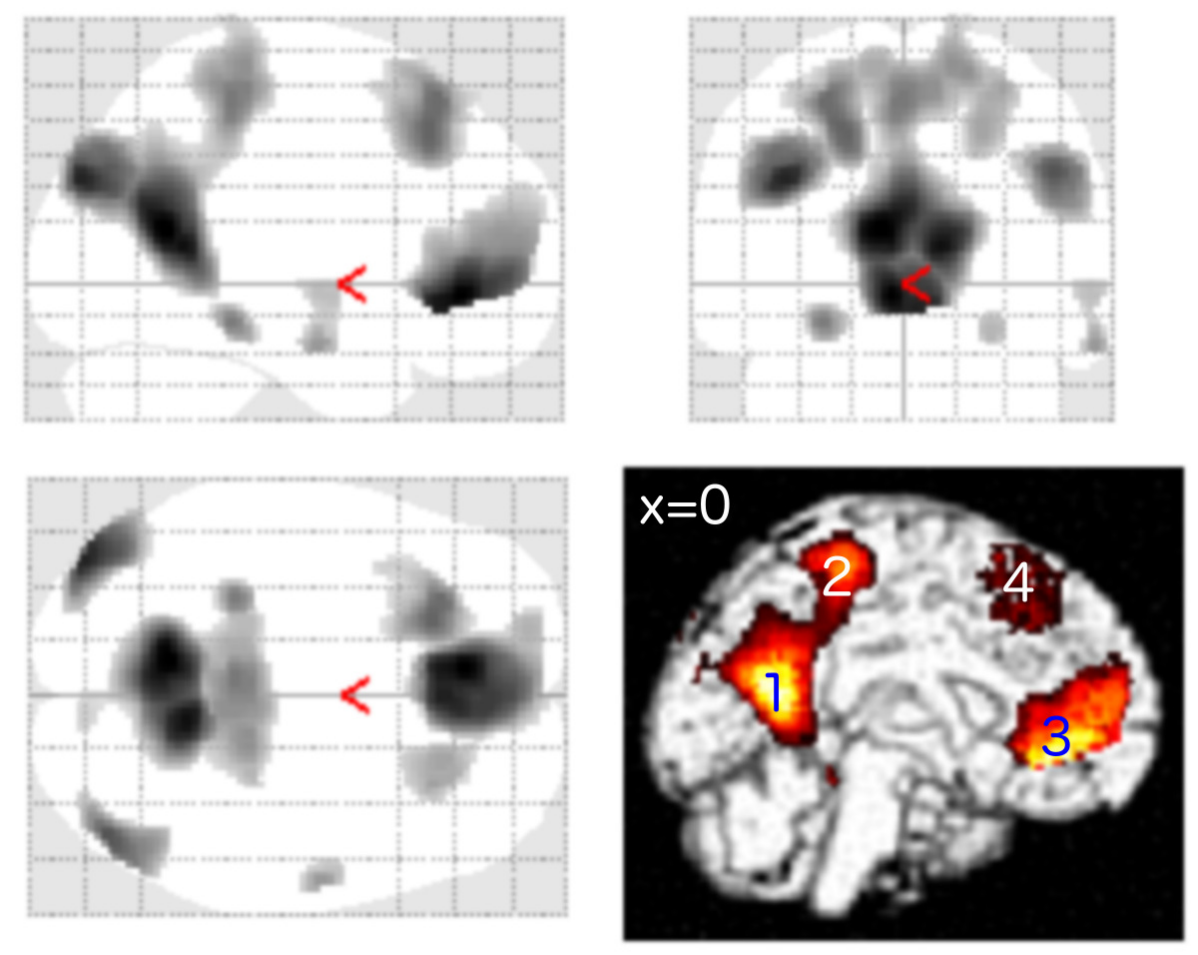
One-sample *t*-test from first fMRI scan of all participants (*n* = 61) contrasting the inter-stimulus interval (ITI) against meaningful words. The figure presents the “glass brain” output of SPM as well as clusters overlaid on the median plane of a T1-weighted brain image. The task-negative condition comprised clusters in LP, PCC (1), precuneus (2), superior parietal lobe, ventro-medial PFC (3), supplemental motor area (4), and dorsolateral prefrontal cortex (*p* < 0.001 uncorrected for visualization; for more details see Table 2). These patterns were closely matched to the Default Mode Network (DMN).

The general task-negative activity was measured by contrasting the brain activity during the ITI period against the brain activity during the word recognition condition using scans from T1. The task-negative activities from all participants (*n* = 61) were compared using a one-sample *t*-test as implemented in SPM. As expected, the DMN network was activated, including MPF, LP, PCC, and PCC/precuneus (Figure 1 and Table 2). The DMN activation has been found highly significant on cluster level [*p* < 0.001, family-wise error (FWE) corrected for multiple comparisons].

**Table 2:** Location of the activated DMN clusters (^**^p < 0.01; ^*^p < 0.05 after FWE correction for multiple comparisons).

**Contrast/location**	**MNI space (x/y/z)**	***Z*-value**	***p* (cluster)**
**General task-negative activation: inter-trial interval > word condition (*n* = 61; *p* < 0.05; FWE corrected; 25 voxels extent threshold)**			
Left precuneus/posterior cingulate cortex	−8/−58/16	>8	<0.001^**^
Right precuneus/posterior cingulate cortex	10/−52/10	>8	
Ventromedial orbitofrontal cortex	−6/38/−6	>8	<0.001^**^
Left postcentral gyrus	−2/−36/56	6.45	<0.001^**^
Right postcentral gyrus	8/−38/58	5.77	
Left superior frontal sulcus	−22/28/46	6.88	<0.001^**^
Right superior frontal sulcus	22/30/46	5.21	<0.001^**^
Left inferior parietal gyrus	−40/−84/30	>8	<0.001^**^
Right inferior parietal gyrus	46/−78/32	7.26	<0.001^**^
Left hippocampus	−28/−36/−14	7.48	<0.001^**^
**TG > PTC; after > before HNMT; masked with general task-negative activation (*n* = 18/21; *p* < 0.001; uncorrected; 25 voxels extent threshold)**			
Precuneus/posterior cingulate cortex	−8/−62/16	4.06	<0.001^**^
Left inferior parietal gyrus	−48/−76/26	3.95	<0.1
Right inferior parietal gyrus	54/−66/28	3.98	n.s.
Left medial frontal gyrus	−8/50/6	3.59	n.s.
Right medial frontal gyrus	10/56/14	3.65	n.s.
**TG > AC; after > before HNMT; masked with general task-negative activation (*n* = 18/22; *p* < 0.001; uncorrected; 25 voxels extent threshold)**			
Precuneus/posterior cingulate cortex	−2/−54/28	4.28	<0.001^**^
Left inferior parietal lobe	−44/−78/28	3.55	n.s.
Precuneus	−6/−50/−8	3.54	n.s.
**TG > AC & TG > PTC; after > before HNMT; masked with gen. task-negative activation (*n* = 18/22 & 18/21; *p* < 0.001; uncorrected; 25 voxels extent threshold)**			
Precuneus/posterior cingulate cortex	−2/−54/26	4.26	<0.001^**^
Left inferior parietal gyrus	−44/−76/26	3.55	n.s.

A flexible factorial model was used to calculate the “group × time” differences of brain activity alterations. Three conditions were of interest: (1) tinnitus-related effect on the DMN, (2) therapy-related effects on the DMN and (3) a possible intersection between therapy-related and tinnitus-related effects.

**Figure 2:**
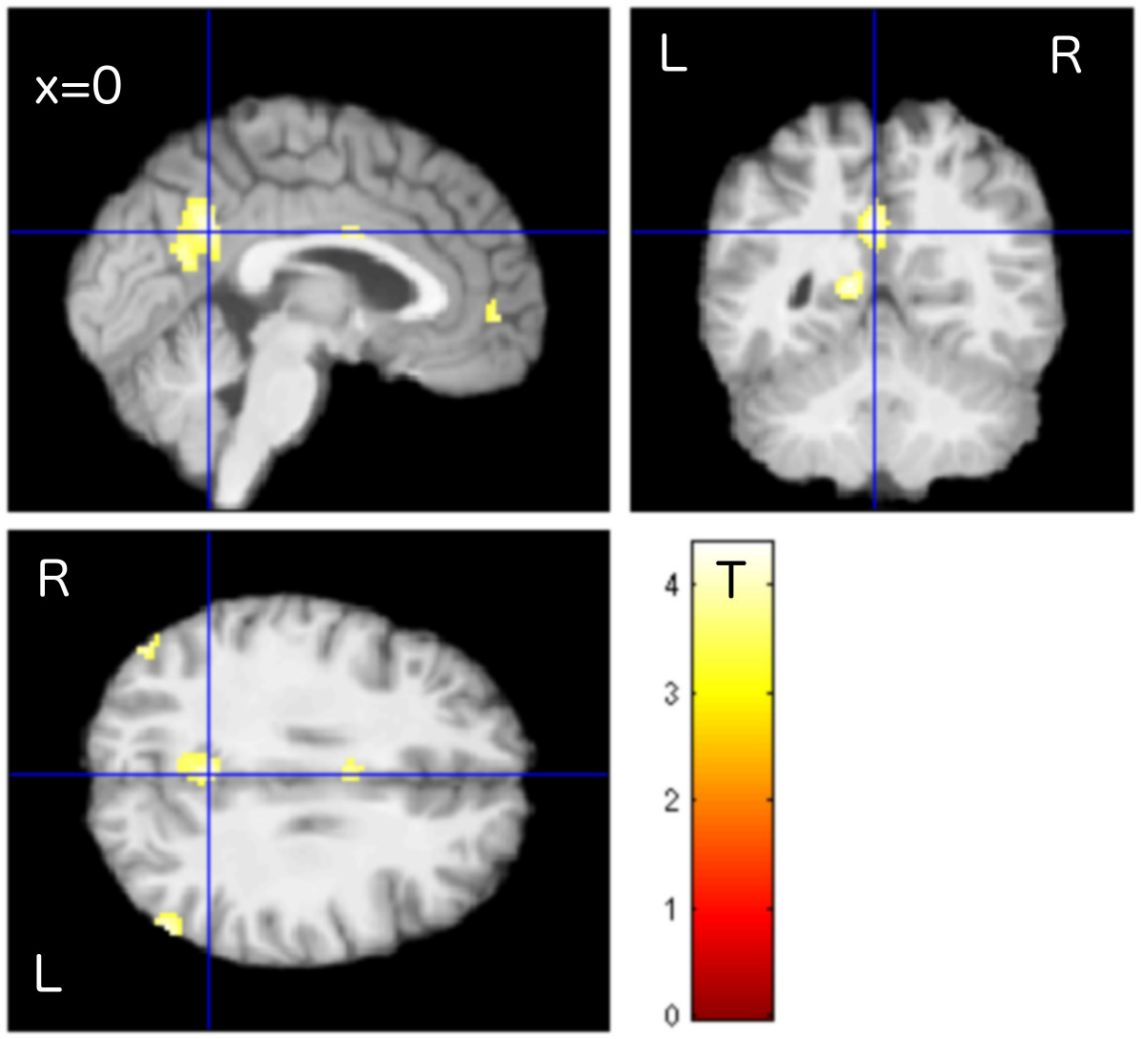
Enhanced activity in the precuneus/PCC region was observed in treated patients (TG) compared to untreated patients (PTC) after the study week. The location of PCC activity by this contrast matched with the DMN pattern as shown in Figure 1.

To assess therapy related effects, the activity change among the tinnitus patients was compared using “group” and “time” as factors of a 2 × 2 ANOVA. The TG group revealed an increase in task-negative activity from T1 to T2, especially in PCC/precuneus, LP, and MPF (Figure 2), while the untreated patients in the PTC group displayed no clusters of enhanced DMN activity in the same time span. The contrast “TG > PTC” was further used as spatial mask (region of interest, ROI) for general therapy-related effects on the DMN.

**Figure 3:**
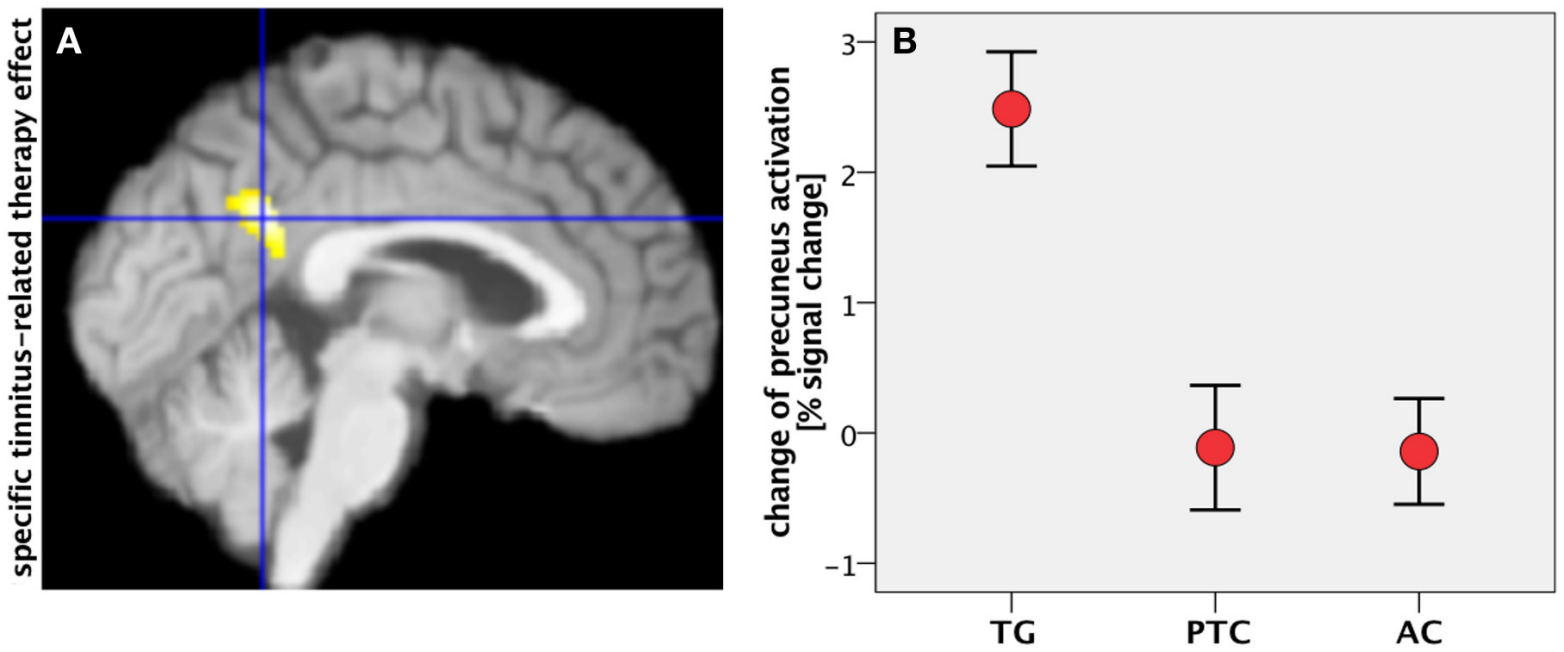
**(A)** Specific tinnitus-related therapy effect by contrasting task-negative activity between treated patients (TG) and “treated” healthy controls (AC) within the mask for therapy-related effects (median plane). **(B)** Change of precuneus activity (in percent signal change) in all groups after the study week. Error bars: standard error of mean.

Tinnitus-related effects were evaluated by comparing the two groups of “treated” participants: the TG and AC groups. Results indicated that the treated tinnitus patients in TG exhibited an increase in the DMN activity compared to the treated healthy participants in AC (Table 2). In contrast to the results from structural data (Krick et al., 2015), there were no HNMT-related effects on DMN in the AC group compared to the PTC group. The individual changes in the PCC/precuneus region were further analyzed by exporting them from SPM and using the statistic tool SPSS (IBM). Scaling the activity change in PCC/precuneus among all participants, the HNMT induced rising activation levels in the TG group solely of 2.5% (Figure 3B). Mann–Whitney *U*-tests revealed significant differences between the TG and PTC groups (*Z* = −3.2; *p* < 0.001) as well as between the TG and AC groups (*Z* = −3.6; *p* < 0.001).

The conjunction of therapy-related and tinnitus-related effects was assessed by contrasting task-negative activity between the TG and AC groups within the spatial mask for therapy-related effects (TG > PTC). It focused on a cluster in the PCC/precuneus area (Figure 3A) and isolated the specific tinnitus-related effect among the therapy-related effects.

### Connection between DMN-activity and tinnitus-distress

**Figure 4:**
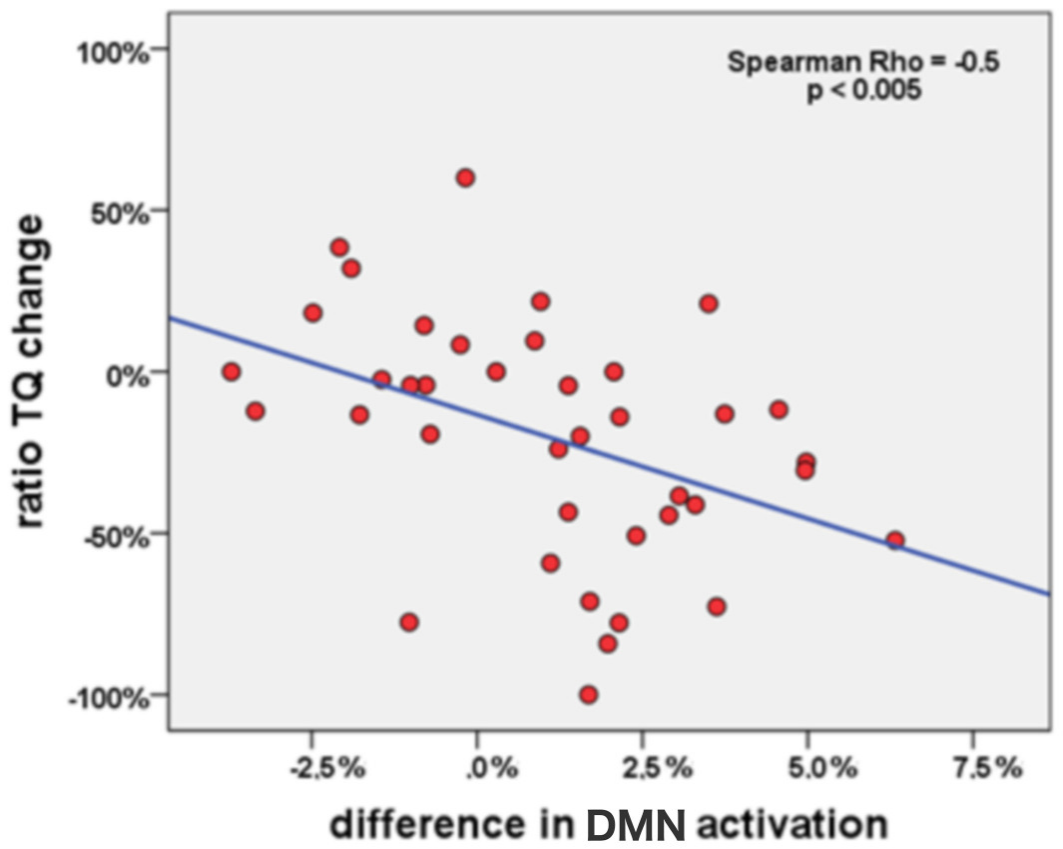
Correlation between change of DMN activation in PCC (percent signal change) and change of tinnitus distress (in percent) in all tinnitus patients (*n* = 39) during the study week.

Using SPSS, the therapy-specific change in the DMN activity was further consolidated by a significant correlation with improvements in tinnitus distress: lower levels of tinnitus distress led to rising DMN activity (*n* = 39; Spearman-Rho: −0.5; *p* < 0.005; Figure 4).

### DMN: functional and structural alterations due to HNMT

**Figure 5:**
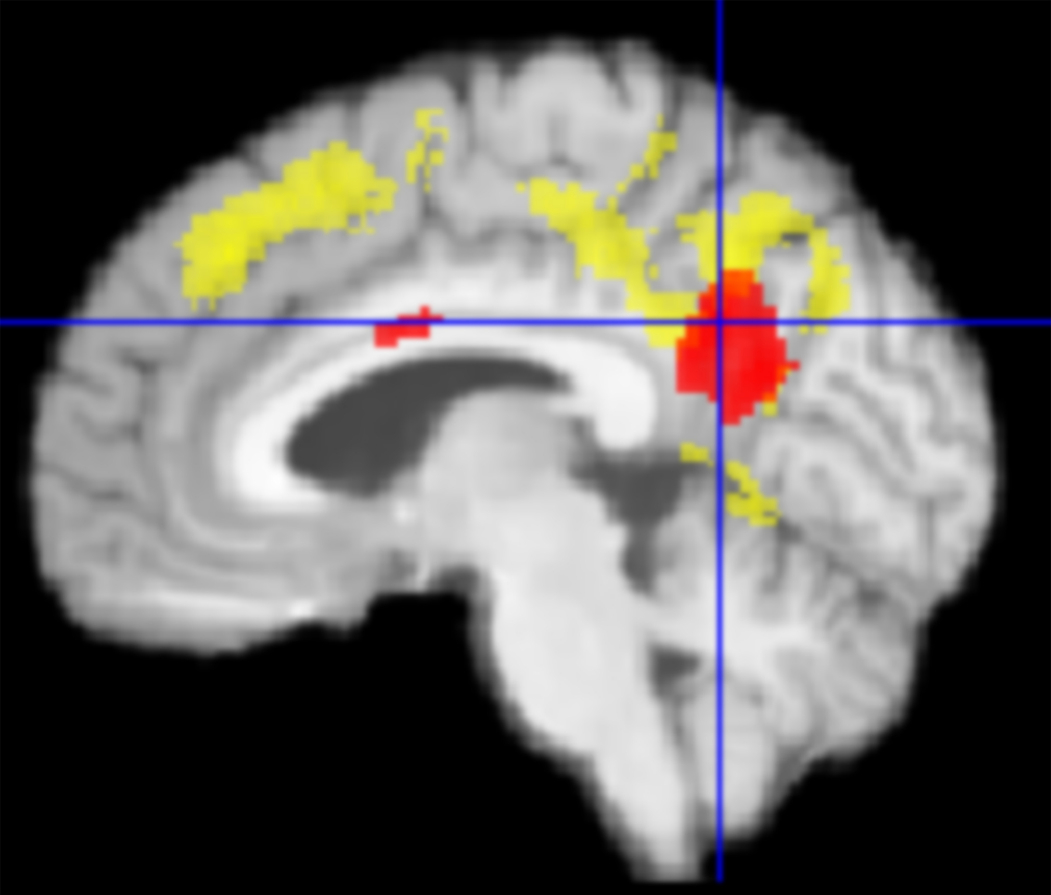
Graphical overlap (median plane) of change in PCC activity (red) und structural gray matter (GM) change from Krick et al. (2015) (yellow) in TG vs. PTC. Both effects converged in the precuneus/PCC-region.

**Figure 6:**
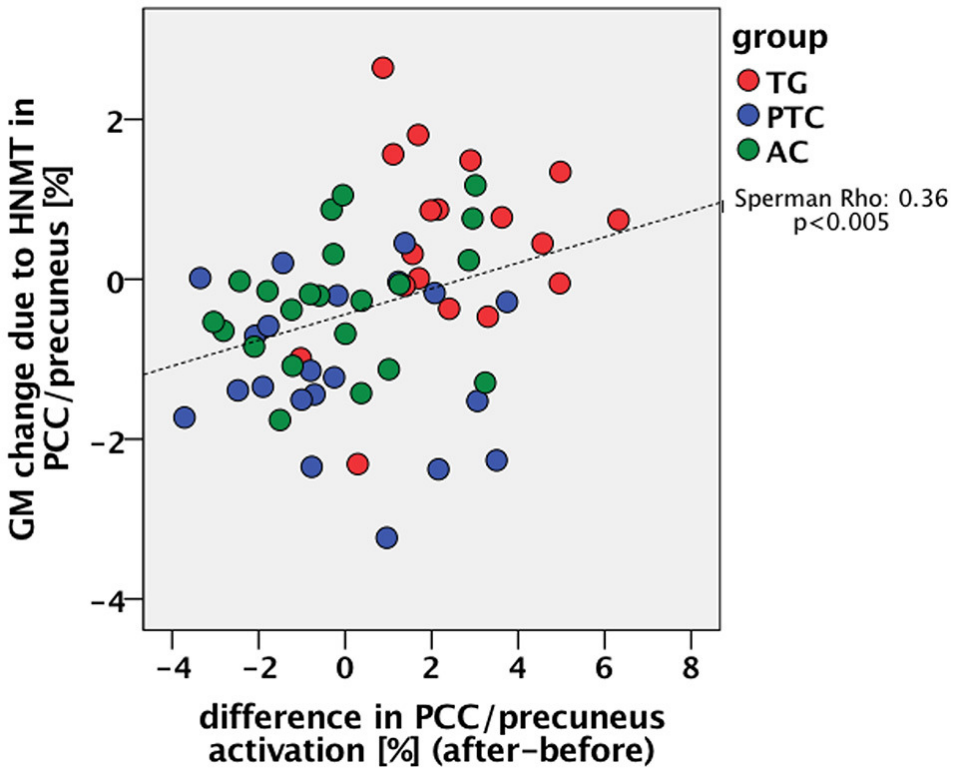
Relation between the previously reported change of GM density in the PCC/precuneus by the HNMT application (Krick et al., 2015) and the measured change of PCC activity.

For this synoptic presentation, structural alterations due to HNMT (Krick et al., 2015) and the functional findings regarding the therapy-related effect reported here were each exported from SPM and analyzed by SPSS. The initial assumption of a general relationship between the DMN activity and the HNMT-induced structural alterations in this network was shown in the correlation between the dynamics of the precuneus activity and structural GM alterations (*n* = 61; Spearman-Rho: 0.36; *p* < 0.005) over the study period (Figure 6). The anatomical convergence of structural and functional effects was able to figure out by an overlap in the precuneus/PCC region (Figure 5).

## Discussion

We used TNAs to observe the DMN in the context of the HNMT tinnitus-therapy control task. The general DMN pattern was similar to findings from previous studies (Fox et al., 2005; Buckner et al., 2008; Raichle, 2015). Since we used twice the identical measurements in the same participants in a longitudinal design, comparable results at T1 and T2 were expected. We used both the HNMT participation (TG vs. PTC) and tinnitus occurrence (TG vs. AC) conditions for analyzing the influence on the DMN activity. Two fMRI measurements were recorded at the beginning and end of the 1-week study period to record longitudinal changes of the BOLD effect. We yielded tinnitus-related effects among general therapy effects due the HNMT approach in the PCC/precuneus region.

The PCC is one of the most interlinked anatomical areas of the brain (Hagmann et al., 2008). Although, its role remains unclear, it seems to play an important role in a variety of cognitive tasks (Leech and Sharp, 2014): The PCC region is involved in internally directed cognition (Buckner et al., 2008). It also seems to control the focus of attention, especially between internally and externally focused thoughts (Leech et al., 2011; Trevis et al., 2016). The PCC region is not homogenous but separated into ventral and dorsal parts (Leech et al., 2011). Regarding both location and behavior of the tinnitus-related therapy effect, the found activation could be assigned to the ventral PCC that in turn is considered to be part of the DMN (Raichle, 2015). The ventral PCC section is strongly connected to the ventral medial PFC as well as the temporal lobes (Choi et al., 2006). The known interplay between ventral PCC and temporal lobes may explain the effect of HNMT on tinnitus, since the structural effects of HNMT showed increase of GM density in temporal areas (Krick et al., 2015).

Lanting et al. (2016) observed suppressed addressability of DMN by TNAs in tinnitus patients in comparison to healthy controls. Ueyama et al. (2013) measured reduced global connectivity in the DMN-specific RSN regions, including the PCC, with increasing loudness of tinnitus. This means that suffering from tinnitus can cause a degradation of the PCC/precuneus functions in task-negative phases (Lanting et al., 2016). Independent component analysis using resting-state fMRI also partly indicated a reduced engagement of this DMN region in tinnitus patients due to a more task-based state due to perception of the internal noise (Schmidt et al., 2013). However, there are contradictory results that showed increased connectivity to the precuneus in pulsatile tinnitus (Han et al., 2015). These relations between tinnitus strength and DMN alterations led to our hypothesis of the HNMT effects on DMN and especially on the PCC/precuneus region.

The modulation of the tinnitus percept by a short-term intervention lasting 5 days only afforded an opportunity to have an insight into the relationship between therapy-induced tinnitus modulation and related DMN activity. The data revealed a rising task-negative activity in tinnitus patients following HNMT, especially in the ventral PCC. The increase of PCC activity was accompanied by rising GM density in this region (Krick et al., 2015). One could assume that HNMT initiated a neural reorganization, reversely to the tinnitus influence. The initial question about the relation between structural GM alterations and their underlying dynamics of brain activity must be resolved toward the convergence between both processes. This effect on tissue level correlated with the clinical therapy effect as measured by TQ. Argstatter et al. (2012) were able to demonstrate the long-term character of clinical therapy success. For this reason, one could assume some kind of neural rehabilitation of the DMN as a result of HNMT.

Since it is known that precuneus and PCC are also involved in the perception of musically induced emotions (Blood et al., 1999) and auditory imagery (Yoo et al., 2001), HNMT incorporates a module aimed at re-conditioning the emotional negative reaction toward the tinnitus percept by using auditory-based mental imagination. Due to the established positive emotional connotation in course of the therapy, the patients usually succeed in decoupling their negative reactions toward the tinnitus sound or even manage to completely ignore the acoustic interference. A reduced activity and a connectivity pattern of the PCC/precuneus region are known to be related to tinnitus distress (Maudoux et al., 2012; Lanting et al., 2016). The tinnitus-reconditioning is extended to tinnitus-prone situations by means of mental imagination. Eventually, patients report less subjective salience of their tinnitus percept. It seems plausible to explain these mechanisms with the activation of an effective noise-cancellation system (Rauschecker et al., 2010).

On the level of auditory processing and attention control, there is evidence that music based stimulation has positive effects on the tinnitus percept. Sound therapies that focus on “notched music listening” distributed lateral inhibition into the tinnitus region and have demonstrated an increase in responsiveness of the PCC (Pape et al., 2014). The “acoustic coordinated reset neuromodulation” (CR; Tass et al., 2012) makes use of computer based auditory stimuli presented as short tones in a random varying sequence above and below the tinnitus frequency and aims at a kind of “anti-kindling” of the underlying neuronal circuits. In CR, a desynchronization of the excitatory connection between PCC and AC has been observed (Tass et al., 2012). Whereas, these approaches are based on passive interventions using computer-modified musical stimuli, HNMT pursues an active involvement by means of repeated stimulation of the central auditory pathway with natural sounds in the range of the individual's tinnitus frequency. Thus, the therapeutic sounds are processed in conscious interaction. Since the PCC/precuneus region has been shown to be involved with the discrimination of musical chords (Fujisawa and Cook, 2011), it seems plausible to attribute some of the changes found in this region to changes in the effect of HNMT due to the corresponding training for frequency discrimination. The harmonic chord perception and the HNMT effect resulted in neighboring activations within the ventral PCC/precuneus region. However, whether the HNMT's effect relies on the explicit perception of frequency relations or emotional relaxation-related effects currently remains an unsolved question.

### Limitations

Functional measurements of brain activity can only depict the relative levels of hemodynamic blood flow between defined conditions, in this case in task-positive and task-negative states. Although, we observed some changes with the interaction of participant group and time, it is safe to say that this cannot originate from TNA. One can only argue that identical tasks performed at two time points may primarily evoke similar responses in all participants. This led to the logical consequence that the task-negative responses might be changed.

Although, the DMN-specific RSN seems to interplay with the so-called “tinnitus core” (Husain and Schmidt, 2014), our findings were not able to depict its relation to the network for tinnitus maintenance itself (Husain, 2016).

## Conclusion

Unlike other auditory procedures, HNMT commits the patients to take an active role in overcoming their tinnitus distress. HNMT aims to target some the neuronal hubs that possibly trigger tinnitus distress. These assumed mechanisms were reflected by a distinct correlation between reduced tinnitus distress and increased DMN activity. As a diminished connectivity to the precuneus seems to be a characteristic pattern for non-pulsatile tinnitus patients (Han et al., 2015; Lanting et al., 2016), we were able to confirm a relationship between the role of PCC/precuneus and the effects of HNMT in recent-onset tinnitus.

## Ethics statement

This study was carried out in accordance with the recommendations of the ethic board of Saarland/Germany with written informed consent from all subjects. All subjects gave written informed consent in accordance with the Declaration of Helsinki. The protocol was approved by the ethic board of Saarland.

## Author contributions

CK: MRI measurements, analysis of MRI data, data interpretation. HA: Development of Heidelberg Neuro-Music Therapy, therapy management, statistics. MG: therapist, analysis of clinical data, statistics. PP: tinnitus diagnostics, clinical therapy control. WR: neuroradiological screening, coordinator between the facilities, study coordinator.

### Conflict of interest statement

The authors declare that the research was conducted in the absence of any commercial or financial relationships that could be construed as a potential conflict of interest.
