# Supplementary material for: Heidelberg Neuro-Music Therapy Enhances Task-Negative Activity in Tinnitus Patients
Source: Front Neurosci. 2017 Jul 7;11:384. doi: 10.3389/fnins.2017.00384 (PMC5500649; doi:10.3389/fnins.2017.00384)
Supplement: Supplementary file 1 [file Presentation1.PDF]

Appendix A: Audiograms of tinnitus patients and healthy controls

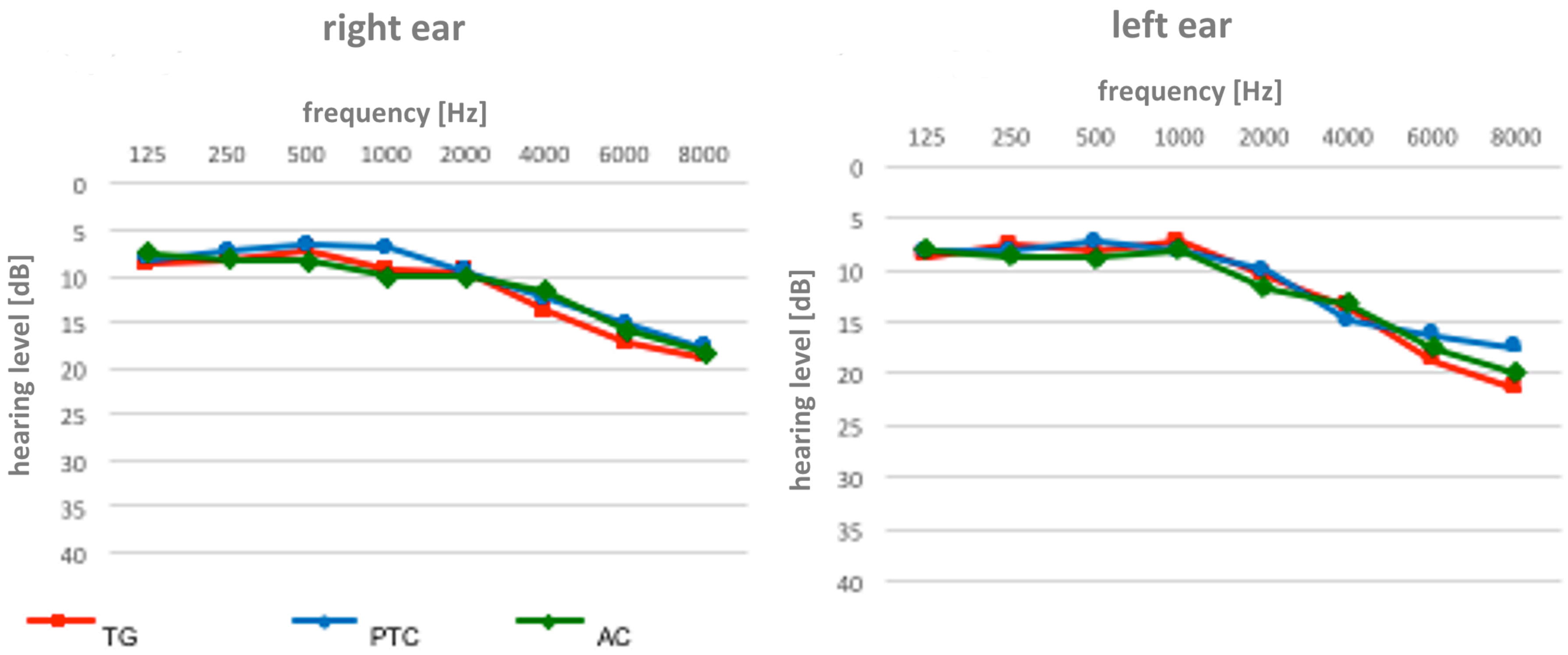

Figure 7: The frequency distributions of audiograms from right and left ears show very similar pattern between patient groups (TG, PTC) and healthy controls (AC).
